# High prevalence of SARS-CoV-2 antibodies and low prevalence of SARS-CoV-2 RNA in cats recently exposed to human cases

**DOI:** 10.1186/s12917-024-04150-4

**Published:** 2024-07-09

**Authors:** Laurence Daigle, Hattaw Khalid, Carl A. Gagnon, Julie Arsenault, Dorothee Bienzle, Sarah-Kim Bisson, Marie-Claude Blais, José Denis-Robichaud, Caroline Forest, Valérie Grenier St-Sauveur, Marika Koszegi, Jennifer MacNicol, Nicolas Nantel-Fortier, Charlotte Nury, Natalie Prystajecky, Erin Fraser, Hélène Carabin, Cécile Aenishaenslin

**Affiliations:** 1 Faculté de médecine vétérinaire, Université de Montréal, Saint-Hyacinthe, Qc Canada; 2 Groupe de recherche en épidémiologie des zoonoses et santé publique (GREZOSP), Faculté de médecine vétérinaire, Université de Montréal, Saint-Hyacinthe, Qc Canada; 3 Centre de recherche en santé publique de l’Université de Montréal et du CIUSSS du Centre-Sud-de-l’Île-de-Montréal, Montréal, Qc Canada; 4 BC Centre for Disease Control, Vancouver, BC Canada; 5 School of Population and Public Health, UBC Centre for Disease Control, University of British Columbia, Vancouver, BC Canada; 6 Swine and Poultry Infectious Diseases Research Center – FRQ, Faculté de médecine vétérinaire, Université de Montréal, Saint-Hyacinthe, Qc Canada; 7 Molecular Diagnostic Laboratory (MDL), Centre de Diagnostic Vétérinaire de l’Université de Montréal (CDVUM), Faculté de médecine vétérinaire, Université de Montréal, Saint-Hyacinthe, Qc Canada; 8 Ontario Veterinary College, University of Guelph, Guelph, ON Canada; 9 Independent Researcher, Amqui, QC Canada; 10 Department of Pathobiology, University of Guelph, Guelph, ON Canada; 11 Department of Pathology and Laboratory Medicine, University of British Columbia, Vancouver, BC Canada

**Keywords:** Cats, COVID-19, Exposure, One Health, Prevalence, SARS-CoV-2, Households

## Abstract

**Background:**

The primary objective of this cross-sectional study, conducted in Québec and Bristish Columbia (Canada) between February 2021 and January 2022, was to measure the prevalence of viral RNA in oronasal and rectal swabs and serum antibodies to severe acute respiratory syndrome coronavirus 2 (SARS-CoV-2) amongst cats living in households with at least one confirmed human case. Secondary objectives included a description of potential risk factors for the presence of SARS-CoV-2 antibodies and an estimation of the association between the presence of viral RNA in swabs as well as SARS-CoV-2 antibodies and clinical signs. Oronasal and rectal swabs and sera were collected from 55 cats from 40 households at most 15 days after a human case confirmation, and at up to two follow-up visits. A RT-qPCR assay and an ELISA were used to detect SARS-CoV-2 RNA in swabs and serum SARS-CoV-2 IgG antibodies, respectively. Prevalence and 95% Bayesian credibility intervals (BCI) were calculated, and associations were evaluated using prevalence ratio and 95% BCI obtained from Bayesian mixed log-binomial models.

**Results:**

Nine (0.16; 95% BCI = 0.08–0.28) and 38 (0.69; 95% BCI = 0.56–0.80) cats had at least one positive RT-qPCR and at least one positive serological test result, respectively. No risk factor was associated with the prevalence of SARS-CoV-2 serum antibodies. The prevalence of clinical signs suggestive of COVID-19 in cats, mainly sneezing, was 2.12 (95% BCI = 1.03–3.98) times higher amongst cats with detectable viral RNA compared to those without.

**Conclusions:**

We showed that cats develop antibodies to SARS-CoV-2 when exposed to recent human cases, but detection of viral RNA on swabs is rare, even when sampling occurs soon after confirmation of a human case. Moreover, cats with detectable levels of virus showed clinical signs more often than cats without signs, which can be useful for the management of such cases.

**Supplementary Information:**

The online version contains supplementary material available at 10.1186/s12917-024-04150-4.

## Background

Coronavirus Disease 2019 (COVID-19) is caused by the severe acute respiratory syndrome coronavirus 2 (SARS-CoV-2), and is responsible for significant morbidity and mortality in humans globally [1]. Following its emergence, the spread of SARS-CoV-2 has been driven by human-to-human transmission [2]. Spillback events, which are subsequent transmissions from humans to other animal species, have been reported in many wild and domestic animal species [3–5].

Cats (*Felis catus*) are susceptible to SARS-CoV-2 infection and constitute a species of particular interest, because they live in close proximity to humans, while also frequently roaming outdoors. Infection in cats has been reported to be often asymptomatic, but also manifests with mild respiratory and gastrointestinal signs of disease [6, 7]. Severe cases are rare and deaths have primarily occurred in animals with existing comorbidities [8]. In experimental infections, viral RNA was detectable for up to two weeks [9, 10], while in the community, studies reported RNA detection for up to four weeks after the household human case confirmation [7, 11, 12].

In studies from multiple countries, SARS-CoV-2 RNA was detected in 8 to 40% of cats recently exposed to the virus in their household, with up to 70% having SARS-CoV-2 serum antibodies [7, 11, 13–21]. Close contacts with an infected human (licking, sleeping in the same bed, being hand fed) [11, 13, 15, 19], and living in households with more than one infected human [17–19] were identified as factors associated with RNA detection and the presence of SARS-CoV-2 antibodies in serum. These studies had sample sizes ranging from eight to 172 cats, and additional data on prevalence and associated risk factors would contribute to strengthening previous findings.

The main objective of this study was to estimate the prevalence of SARS-CoV-2 RNA and the presence of SARS-CoV-2 antibodies in serum amongst cats sampled shortly after the diagnosis of one or more humans in the household. Secondary objectives were to explore cat- and household-level risk factors associated with cats’ seropositivity, and to evaluate if cats’ status for RNA detection or seropositivity to SARS-CoV-2 antibodies was associated with clinical signs compatible with COVID-19.

## Methods

This cross-sectional study was conducted in the provinces of Québec (Qc) and British-Columbia (BC) in Canada. All procedures were approved by the research ethics committee of the Université de Montréal and the University of British Columbia, for humans (Qc: certificate CERSES-20-149-D; BC: certificate #H20-03452), and for animals (Qc: certificate CEUA 20-Rech-2091; BC: certificate A20-0268-A002). This study is reported as per the Strengthening the Reporting of Observational Studies in Epidemiology – veterinary (STROBE-Vet) guidelines (Supplementary material: Checklist – S1).

### Recruitment and visits

The sample size was limited to a maximum of 60 households and 80 cats. Households with one or more laboratory-confirmed human COVID-19 cases and where one or more cats lived were recruited on a voluntary basis from three sources: (1) households participating in a joint BC-Qc human health household transmission study; (2) media and social media advertisement where interested households were invited to fill an online questionnaire which was used to validate the inclusion criteria; or (3) households identified by local public health authorities of the Montérégie region (Direction de la santé publique de la Montégérie; DSP) in Québec. Households from these sources were contacted by the research team via phone to schedule a first visit. The target interval between the date of laboratory confirmation of the household’s index case (i.e., first infected household member) and the first sampling visit was of a maximum of seven days and human SARS-CoV-2 case(s) in the household had to be managed at home at the time of recruitment for a household to deemed eligible for inclusion. One or more adult in the household had given consent to participate in study, and the enrollment of cats was done only after obtaining the owner’s informed consent. Due to challenging recruitment in BC, two cats were sampled between eight and 15 days after the household index’s laboratory confirmation date.

A longitudinal component was included in the study whereas cats were re-sampled one to three weeks after the first visit. If viral RNA was still detectable at the second visit, a third visit was done two to three weeks later.

### Animal sampling and testing

At most three cats aged at least six months old, that could be handled, and stayed inside at least 50% of the time were recruited from consenting households. Sampling consisted of a combined nasal and oropharyngeal (NOP) swab, a rectal swab, and a serum sample. To ensure the well-being of the cats during the procedure, only cooperative cats in the household were selected. The procedures were carried out by a veterinarian and an animal health technician, who first assessed the restraint needs to ensure the comfort of the animal. The sampling procedures lasted a few minutes.

Two different protocols were used in the different provinces, because at the time of the study, these were the only two RT-qPCR protocols available to test animal samples in Québec and in British Columbia. Herein a brief description of the methods used by laboratories of both provinces.

Swabs collected in Québec were analyzed by the Molecular Diagnostic Laboratory of the Centre de diagnostic vétérinaire de l’Université de Montréal (Saint-Hyacinthe, Canada). Swabs were kept at 4 °C and were subsequently analyzed for the presence of SARS-CoV-2 viral RNA using RT-qPCR between one to five days after sampling. The samples were initially treated to inactivate infectious virion particles using a heat inactivation step of 2 min at 90 °C, prior to nucleic acid extraction. Afterwards, the viral genome was extracted according to laboratory’s standard operating procedures followed by the detection of the viral genome using a SARS-CoV-2 RT-qPCR assay. Briefly, the two genome extraction methods were adapted from two commercial kits, the QIAamp cador Pathogen Mini kit (QIAGEN, Mississauga, ON, Canada) and BioSprint 96 One-For-All Vet kit (QIAGEN), using a KingFisher automated magnetic beads system (Thermo-Fisher Scientific, Ottawa, ON, Canada), and a Qiacube automated filtration-based system (QIAGEN), respectively. In resume, all reagents of both kits were acquired in bulk and combined to extract the viral genome from the clinical samples. The SARS-CoV-2 RT-qPCR assay was composed of two qPCR reactions, one targeting the N viral gene (protocol PR-BM-131), while the other was targeting the E viral gene. The N viral gene qPCR detection was based on a detection method developed and kindly provided by Dr Hugues Charest from the Institut national de santé publique du Québec (INSPQ), while the E viral gene qPCR detection method was adapted from a previous report [22].

Swabs collected in British Columbia were analyzed at the British Columbia Centre for Disease Control Public Health Laboratory (Vancouver, Canada). Viral nucleic acid was extracted using the ThermoFisher’s Viral RNA kit on the Kingfisher Flex/MagMAX systems (Thermo-Fisher Scientific, Ottawa, ON, Canada). A multiplex RT-qPCR method developed for clinical detection was used for SARS-CoV-2 detection. This method targets the RdRP gene [23] and E gene [22].

Sera were analyzed for the detection of cat IgG antibodies to SARS-CoV-2 spike protein (SARS-CoV-2 S1, GenScript, Piscataway, NJ), using enzyme-linked immunosorbent assays (ELISA). All sera samples were kept stored at -20 °C until they were sent to the Bienzle Research Laboratory at the University of Guelph (Canada). Briefly, absorption immunoassay plates (96-well, ThermoFisher, Mississauga, ON) were coated overnight at 4 °C with 2 µg/mL of His-tagged SARS-CoV-2 S1 (GenScript). The following day, wells were washed 3x, blocked with 3% skim milk in Tris buffer for one hour, washed 3x, and then 60 µL of five 3-fold dilutions (1:100, 1:300, 1:900, 1:2,700 and 1:8,100) of each serum sample was added. Plates were incubated for two hours, washed 3x, and secondary antibodies (goat anti-cat IgG; Abcam, Waltham, MA) conjugated to horseradish peroxidase (HRP) and diluted 1:5,000 were added for one hour. Wells were washed 3x, and HRP activity was visualized by adding trimethyl benzidine substrate. Reactions were stopped with sulfuric acid, and optical density (OD) at 450 nm was read. Control samples consisted of serum from a SARS-CoV-2 experimentally-infected cat (kindly provided by Y. Kawaoka, Madison, WI; positive feline control, used at 1:5,000 in ELISA), and negative controls came from three different batches of pooled cat serum from 2016 to 2017, two serum samples from cats with feline infectious peritonitis, and one serum sample from a cat with osteomyelitis and hyperglobulinemia. Each ELISA plate included 16 wells that were not coated with recombinant protein (blank), five replicates 1:100 dilutions of species-specific negative control samples, and five 3-fold dilutions of the positive control and test samples, starting at a 1:100. Samples with an OD > 3 SD from the mean of the negative controls were considered positive, and a subset of these were also assessed with a surrogate virus neutralization assay (cPass, GenScript). The surrogate virus neutralization assay measures blocking of the interaction of the viral receptor binding domain with the ACE2 receptor, and is therefore suitable to measure neutralizing antibodies in a species-independent manner.

### Survey

Questionnaires (Supplementary material: Questionnaires – S2) were developed in French and English by the research team using a previously developed questionnaire (BC Centre for Disease Control - BCCDC) that included questions on cat- and household-level characteristics and management and cats’ clinical signs. All questions were asked at the first visit (Qc: 34; BC: 30), and questions that could change with time were asked during the follow-up visits (Qc: 16; BC: 20). The question about owners’ toilet access was not included in the BC questionnaire. The questionnaires were filled over the phone with the owner following each visit.

### Variables

**Outcomes**. The detection of SARS-CoV-2 RNA was defined as at least one swab (NOP or rectal) with a Ct values ≤ 35 for both tested genes (Qc: N and E genes; BC: RdRP and E genes). Seropositivity to SARS-CoV-2 antibodies was defined as OD > 3 SD above the mean of the negative controls in the ELISA. The presence of clinical signs compatible with COVID-19, used as a secondary outcome, was defined as at least one of the following: coughing, sneezing, nasal discharge, breathing difficulty, weakness or lethargy, vomiting, or diarrhea.

**Exposure**. Cat-level variables were the cat’s age (categories: < 2 years old, 2 to 8 years old, and > 8 years old), sex (female or male), breed (domestic or purebred), and chronic diseases status (presence or absence of diabetes, feline immunodeficiency virus and/or feline leukemia virus infections). It also included if the cat had access to the outdoor or to the owner’s toilet, if the cat was restricted to a specific area of household, and if the cat had been manipulated using personal protective equipment (PPE) since the diagnosis of the index case. Finally, the number of hours spent in close contact with humans in the week prior to the diagnosis of the index case was used (categories: < 2 h, 2 to 21 h, and > 21 h). Household-level variables were the province, the number of people living in the household (categories: 1 or 2, and ≥ 3), the number of people positive to COVID-19 (categories: 1, and ≥ 2), the presence of clinical signs in humans, the number of cats (categories: 1, and ≥ 2), and the time between the diagnosis of the index case and the first sampling visit by our research team (categories: < 4 days, and ≥ 4 days). For the presence of cat’s clinical signs, SARS-CoV-2 RNA detection and seropositivity to SARS-CoV-2 antibodies were considered as exposure variables.

### Statistical analysis

Analyses were conducted in R (version 4.3.0; [24] with the RStudio interface (version 2021.09.0 + 351)). The prevalence proportions and 95% Bayesian credible intervals (BCI) of viral RNA detection and presence of serum antibodies to SARS-CoV-2 were calculated by province for each visit at the cat-level and at the household-level (at least one positive cat; propCI package; [25]). Prevalence ratios (PR) and 95% BCI of seropositivity to SARS-CoV-2 antibodies were calculated for each cat-level and household-level exposure variable, and the PR of clinical signs in cats was calculated for the cats’ PCR status and seropositivity, all using Bayesian mixed log-binomial models that included household as a random intercept (R2jags package; [26, 27]). Such models have a cluster specific interpretation, which was adjusted for household-level exposures by applying a shrinkage factor to the estimates to obtain population average effects [28]. In case of missing values, complete-case analyses were conducted. No multivariable model was run due to the limited sample size.

For all models, weakly informative priors were used. Briefly, priors for the intercepts and the *β*s were normal distributions centered on 0 with sigma = 0.1, and the hyper distribution for the random-effect intercept was normal distribution centered on 0 with sigma following a uniform distribution (Inf,0.0001). Models were assessed in three chains of 100,000 iterations after a burn-in of 50,000 iterations, using a thinning factor of 2. Convergence and autocorrelation were assessed visually, and the effective sample size for each parameter was calculated, with a minimum of 1,000 being considered acceptable [29]. For continuous exposure variables, residuals were inspected visually, but the small sample size yielded suboptimal residuals and categorized models were also assessed.

## Results

**Fig. 1:**
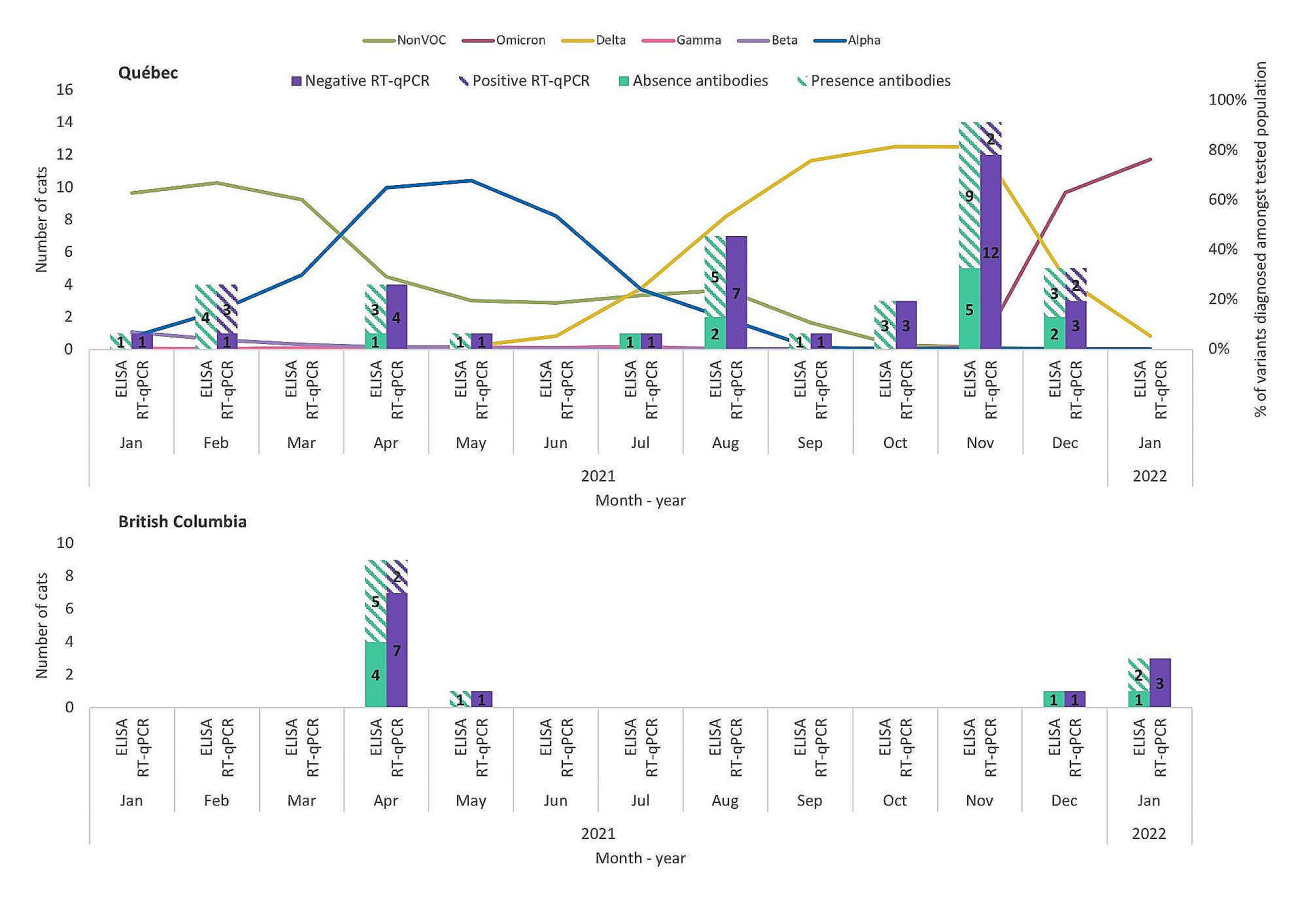
Distribution of recruited cats and virus variants circulating in population at the time of study. Distribution of recruited cats, including serology (green) and RT-qPCR (purple) results, and virus variants circulating in the Québec human population between January 2021 and January 2022 [31]. Sequences circulating in the different provinces were similar. The four types of virus variants circulating in Canada were (1) those considered “not of concern” (green line), (2) Alpha (blue line), (3) Delta (yellow line) and, at the end of our recruitment period, (4) Omicron (dark pink line) [30]

A total of 120 households (Qc: 73; BC: 47) were identified, of which 40 (Qc: 31; BC: 9) were recruited. Non-participation was due to refusals (Qc: 14, BC: 1), answers provided after the delay since the confirmation of the index case (Qc: 4, BC: 0), ownership of an aggressive or outdoor cat (Qc: 2, BC: 1), no answer when called (Qc: 5, BC: 25), or not called due to insufficient resources (Qc: 17, BC: 20). A total of 55 cats were sampled (Qc: 41, BC: 14), with one, two, or three cats sampled per household in 29, seven and four households, respectively. Cats were sampled during four periods: February 2021, April 2021, August 2021, and November 2021 to January 2022, during which the four types of virus variants circulating in Canada were (1) those considered “not of concern”, (2) Alpha, (3) Delta and, starting in December 2022, (4) Omicron [30]. Figure 1 illustrates the distribution of recruited cats, including RT-qPCR and serology results, and virus variants circulating in Québec population at the time of study. Cats were mainly domestic breed (91%), 51% were female, and their age was distributed between < 2 years old (16%), 2 to 8 years old (44%), and > 8 years old (40%; Table 1). People had 0 to 105 h (median = 5) of close contact with cats in the week prior to the diagnosis of the index case. One to five (median = 2.5) people and one to four cats (median = 1) resided in the participating households. 55% of households had more than one person who tested positive to SARS-CoV-2, and 95% had at least one symptomatic person (Table 2).

**Table 1:** Distribution of cat-level characteristics and management of cats amongst the sampled cat population

	Missing values		Seropositivity to SARS-CoV-2 antibodies	
Exposure		*n* cats total (%)	*n* positive cats (%)	PR (95% BCI)
Age	0			
< 2 years old		9 (16%)	6 (66%)	Ref.
2 to 8 years old		24 (44%)	15 (63%)	1.00 (0.59–2.07)
> 8 years old		22 (40%)	17 (77%)	1.19 (0.75–2.41)
Sex	0			
Female		28 (51%)	20 (71%)	Ref.
Male		27 (49%)	18 (67%)	0.93 (0.64–1.32)
Breed	0			
Domestic		50 (91%)	34 (68%)	Ref.
Purebred†		5 (9%)	4 (80%)	0.99 (0.41–1.50)
Chronic disease(s)	0			
No		51 (93%)	34 (67%)	Ref.
Yes		4 (7%)	4 (100%)	1.17 (0.55–1.68)
Access to outdoor	0			
No		41 (75%)	29 (71%)	Ref.
Yes		14 (25%)	9 (64%)	0.88 (0.50–1.29)
Isolation of the cat from the infected human	0			
No		50 (91%)	35 (70%)	Ref.
Yes		5 (9%)	3 (60%)	0.78 (0.24–1.40)
Use of PPE when interacting with the cat	3			
No		47 (90%)	32 (68%)	Ref.
Yes		5 (10%)	3 (60%)	0.80 (0.25–1.43)
Access to owner’s toilet	14			
No		16 (39%)	14 (88%)	Ref.
Yes		25 (61%)	16 (64%)	0.78 (0.52–1.14)
Close contact with the infected human per week	14			
≤ 2 h		12 (29%)	8 (67%)	Ref.
> 2 to < 21 h		18 (44%)	14 (78%)	1.16 (0.74–2.09)
≥ 21 h		11 (27%)	8 (73%)	1.08 (0.59-2.00)

PPE: personal protective equipment

†Bengal (*n* = 1), Himalayan Blue Point (*n* = 1), Siamese (*n* = 2), and Siberian (*n* = 1)

Distribution of cat-level characteristics and management of 55 cats from 40 households in Québec and British Columbia (Canada) with at least one confirmed human case of COVID-19 within 15 days prior to sampling. Prevalence ratio (PR) and 95% Bayesian credibility intervals (BCI) of SARS-CoV-2 seropositivity to SARS-CoV-2 antibodies for each cat-level exposure was calculated using a Bayesian mixed log-binomial models that included household as a random intercept

**Table 2:** Distribution of household-level characteristics and management of cats in the sampled cat population

	*n* households total (%)	*n* cats total (%)	Seropositivity to SARS-CoV-2 antibodies	
Exposure			*n* positive cats (%)	PR (95% BCI)
Province				
Québec	31 (78%)	41 (75%)	30 (73%)	Ref.
British Columbia	9 (22%)	14 (25%)	8 (57%)	0.78 (0.43–1.19)
Number of people				
1 or 2	20 (50%)	25 (45%)	15 (60%)	Ref.
≥ 3	20 (50%)	30 (55%)	23 (77%)	1.24 (0.87–1.89)
Number of people with COVID-19				
1	18 (45%)	26 (47%)	18 (69%)	Ref.
≥ 2	22 (55%)	29 (53%)	20 (69%)	0.99 (0.69–1.43)
Clinical signs in humans				
No	2 (5%)	2 (4%)	2 (100%)	Ref.
Yes	38 (95%)	53 (96%)	36 (68%)	0.98 (0.60–3.72)
Number of cats				
1	27 (68%)	27 (49%)	18 (67%)	Ref.
≥ 2	13 (32%)	28 (51%)	20 (71%)	1.09 (0.76–1.60)
Time between index case diagnosis and first visit†				
< 4 days	13 (32%)	18 (33%)	8 (44%)	Ref.
≥ 4 days	27 (68%)	37 (67%)	25 (68%)	1.47 (0.90–2.79)

†The outcome for this variable was seropositivity at the first visit only

Distribution of household-level characteristics and management of 55 cats from 40 households in Québec and British Columbia (Canada) with at least one confirmed human case of COVID-19 within 15 days prior to sampling. Prevalence ratio (PR) and 95% Bayesian credibility intervals (BCI) of seropositivity to SARS-CoV-2 antibodies for each cat-level exposure was calculated using a Bayesian mixed log-binomial models that included household as a random intercept, and adjusted with a shrinkage factor to the estimates to obtain population average effects. There is no missing values

**Fig. 2:**
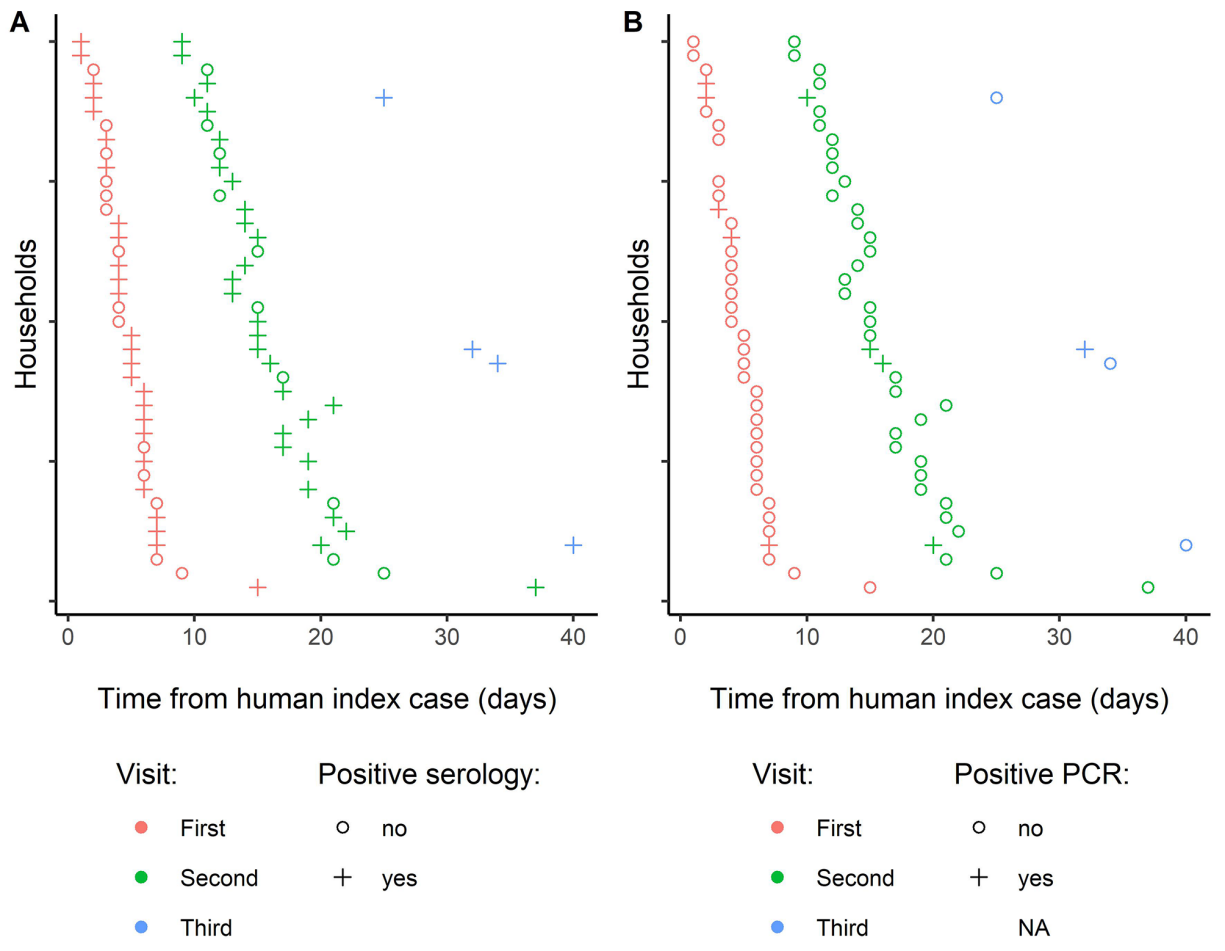
Timeline of the household-level sampling and SARS-CoV-2 antibody and RNA detection test results. Sampling and test results timeline for participating households showing (**A**) results of the antibody to SARS-CoV-2 detection serological test and (**B**) results of the viral RNA detection test. The figure shows the results of all cats sampled in each household at the first, second, and third visit

All households were visited twice, and four were visited a third time. The time between the diagnosis of the index case and the first visit varied between one and 15 days (median = 4), between the first visit and the second visit varied between five and nine days (median = 7), and between the second visit and the third visit was seven days (Fig. 2).

### Detectable viral RNA and presence of SARS-CoV-2 antibodies

A total of nine cats (0.16; 95% BCI = 0.08–0.28) had at least one positive RT-qPCR, all from NOP swabs sampled at the first or second visit. Six cats had detectable viral RNA at the first visit, of which two still had detectable levels at the second visit. Three showed detectable viral RNA for the first time at the second visit. Only one cat of the five tested still had detectable viral RNA at the third visit. In Québec, Ct values for the N gene varied between 25.2 and 34.7 (median = 32.1; *n* = 10) while that for the E gene ranged from 25.8 to 34.7 (median = 32.6; *n* = 12). In British Columbia, Ct values for the RdRP gene varied from 19.2 to 34.9 (median = 24.0; *n* = 3) and for the E gene were 19.4 and 24.2 (*n* = 2). Of the cats considered negative to the RT-qPCR, three (2 NOP and 1 rectal samples) had a positive result for one of the tested genes, but a negative result for the other. No clear trend was noted amongst the three cats testing positive at least twice in terms of change in Ct values.

**Fig. 3:**
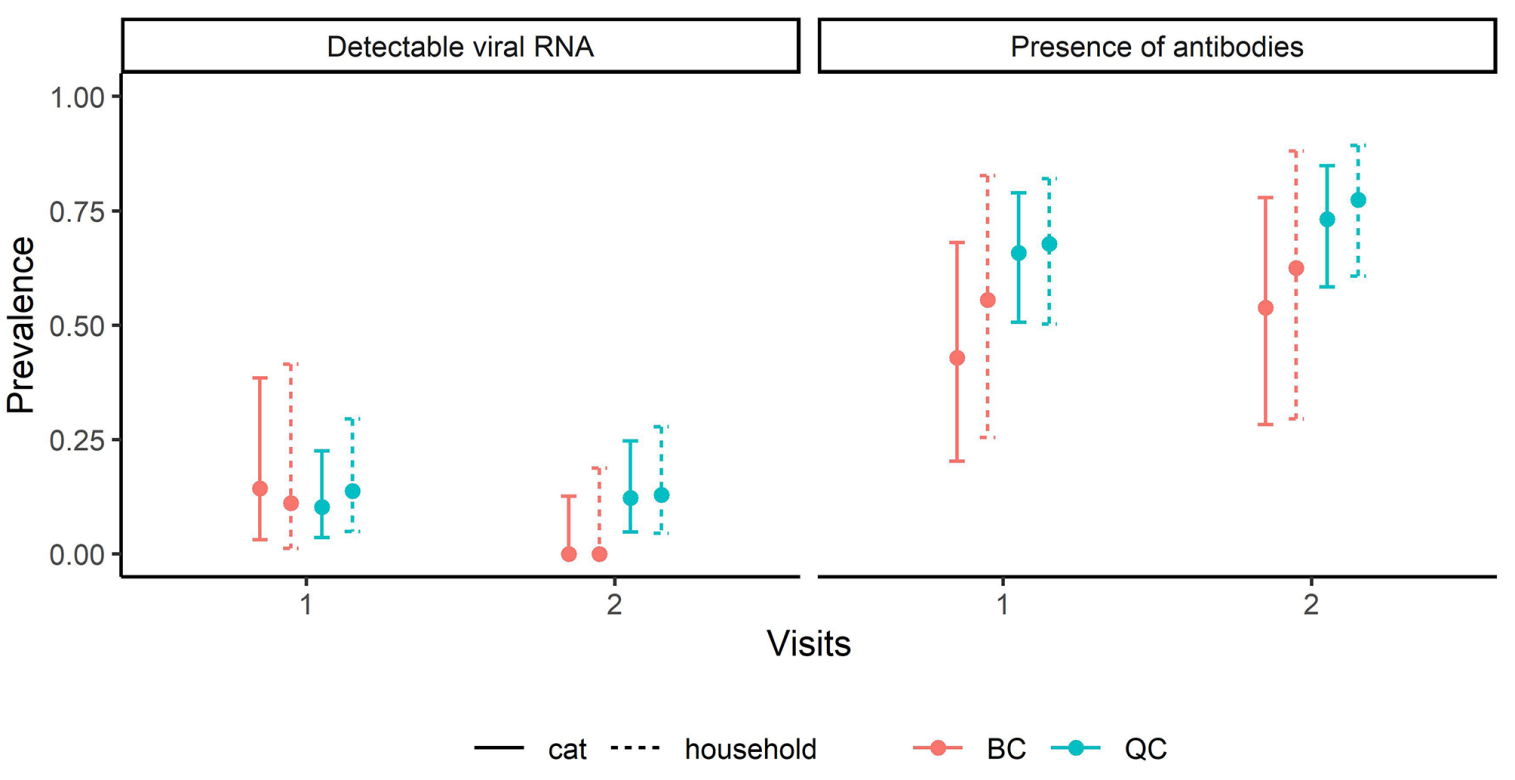
Prevalence of detectable viral RNA or antibodies to SARS-CoV-2 at the cat- and household-levels. Prevalence and 95% credibility intervals of cats (*n* = 55) and of households (*n* = 40) having at least one cat with detectable viral RNA or seropositive for antibodies to SARS-CoV-2 in Québec or British Columbia (Canada), at a first visit (within 15 days with at least one confirmed human case of COVID-19) or a second visit (8 to 22 days following the first visit)

A total of 38 cats (0.69; 95% BCI = 0.56–0.80) had at least one serum sample with SARS-CoV-2 antibodies: 33 were positive at the first visit, of which 32 still had a positive serology at the second visit, and five became seropositive between the first and second visits. Seven out of eight seropositive samples also had SARS-CoV-2 neutralizing antibodies. All cats with at least one positive RT-qPCR sample had at least one serum sample with SARS-CoV-2 antibodies. The prevalence of cats positive to the RT-qPCR or serology did not differ between provinces and visits (Fig. 3). When considering households as the unit, prevalence was similar between cat- and household-levels.

None of the risk factors tested, at the cat- and household-levels, showed an association with the presence of SARS-CoV-2 antibodies in serum (95% BCI included 1; Tables 1 and 2). However, the strongest magnitude of association with the presence of SARS-CoV-2 antibodies was found for cats sampled four days or more after the diagnosis of the human index case compared to less than four days after (PR = 1.47; 95% BCI = 0.90–2.79). It was only estimated for the first and second visits, since very few cats were included for a third visit and only one of the five sampled cats still had detectable viral RNA at this visit.

### Clinical signs

**Fig. 4:**
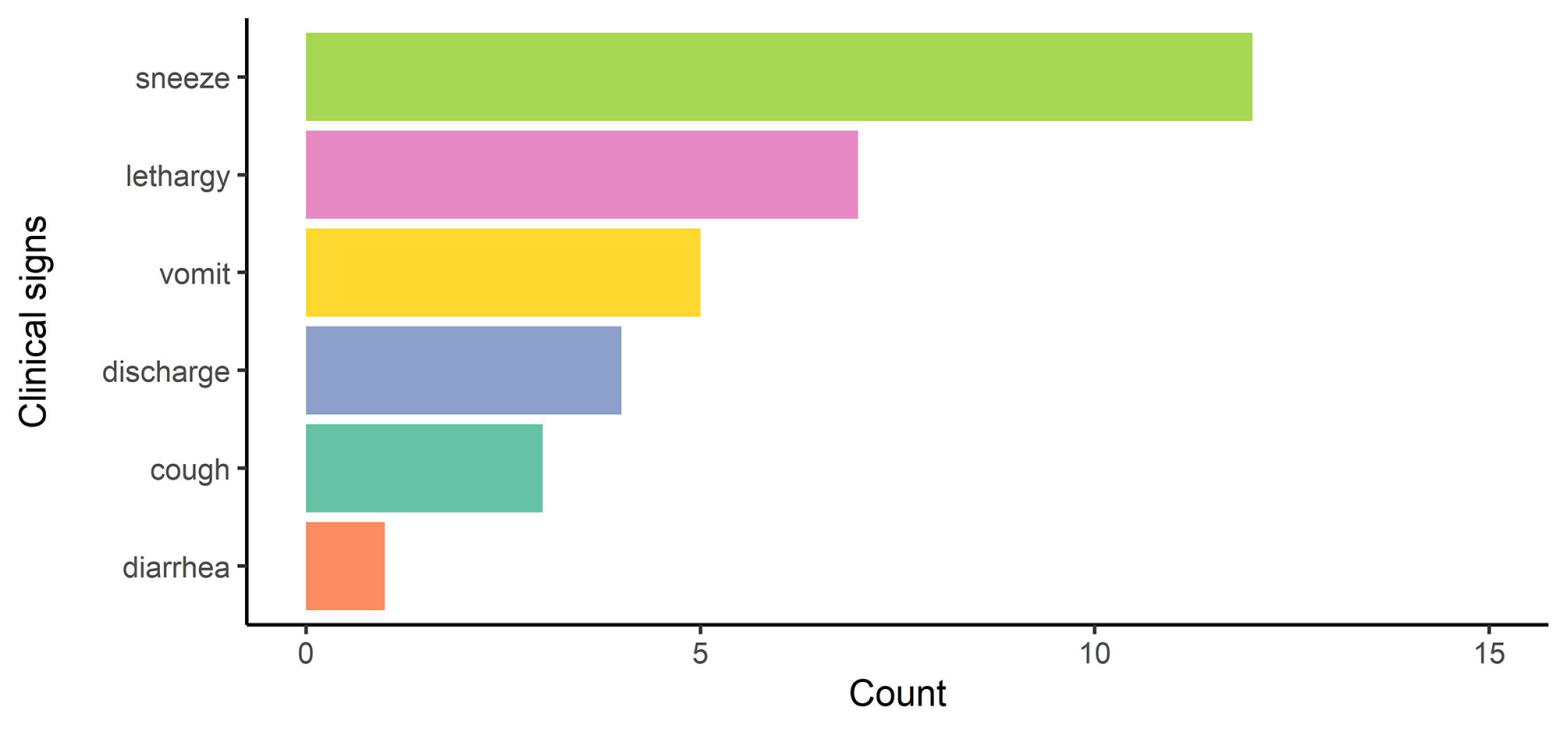
Distribution of clinical signs compatible with COVID-19 amongst 21 symptomatic cats. Distribution of clinical signs amongst the 21 cats that had one (11/21; 52%) or more (10/21; 48%) clinical signs compatible with COVID-19, including 16 cats seropositive to SARS-CoV-2 antibodies (16/38; 42%), and in five seronegative cats (5/17; 29%)

Owners of 21 cats (0.38; 95% BCI = 0.26–0.51) reported one (*n* = 12), two (*n* = 7), or three (*n* = 2) clinical signs, the most frequent sign being sneezing (Fig. 4). At least one clinical sign was identified in 16 cats seropositive to SARS-CoV-2 antibodies (16/38; 42%), and in five seronegative cats (5/17; 29%). At least one clinical sign was identified in seven cats with detectable viral RNA (7/9; 78%), and in 14 cats without detectable viral RNA (14/46; 30%). While seropositivity to SARS-CoV-2 antibodies was not associated with the presence of one or more clinical sign (PR = 1.35; 95% BCI = 0.63–3.52), the prevalence of cats with at least one clinical sign was 2.12 (95% BCI = 1.03–3.98) times higher amongst cats with detectable viral RNA compared to those without.

## Discussion

The prevalence values of 16% for detectable SARS-CoV-2 RNA and of 69% for seropositivity to SARS-CoV-2 antibodies in cats exposed to confirmed human cases in their household found in this study are similar to previously reported studies that used a similar design (8 to 40% of RNA detected and up to 70% having serum SARS-CoV-2 antibodies), except for the timing of sampling [7, 11, 13, 14, 19–21]. While most of these previous studies sampled cats three to 15 days after the diagnosis of the index case, the delay was longer, i.e. up to 29 days on average, in a few studies [11, 17, 21]. The prevalence of viral RNA detection was lower (0 to 8%) in studies with longer delays, suggesting a shorter period of detectable viral excretion in some cats. While the prevalence of detectable viral RNA in the present study decreased between the first and second visit, the difference was not major. Moreover, one of the five cats sampled at the third visit (32 days after the diagnosis of the human index case) had detectable viral RNA. This suggests that some cats have detectable viral RNA for longer periods of time, as suggested by Bienzle et al. [11], although this would need to be confirmed using larger sample sizes.

The prevalence of seropositivity in the different studies could also be affected by the sampling timing due to the time required to develop SARS-CoV-2 antibodies. In the present study, there was no difference in SARS-CoV-2 seroprevalence between the two visits, although the seroprevalence was lower at the first visit if sampling was done less than four days after the confirmation of the household’s index case. This is in line with a SARS-CoV-2 challenge study that found some cats developed SARS-CoV-2 antibodies as early as five days post-exposure, but it was more common from seven days post-exposure [9].

We did not find associations between cat- and household-level exposure variables and SARS-CoV-2 seropositivity, likely due to the absence of very strong associations and our small sample size. Previous studies, with similar populations and sample size between eight and 172 cats, found management risk factors for the detection of SARS-CoV-2 RNA and the presence of SARS-CoV-2 antibodies, but the magnitudes of effect were stronger than in our study [11, 13, 15, 18, 19]. For example, sleeping in the owner’s bed was positively associated with the presence of SARS-CoV-2 antibodies (odds ratio (OR) = 5.8; 95% CI = 1.1–29.4) [11] and with viral RNA detection (OR = 17.2; 95% CI = 3.2-188.6) [15], and households with multiple people with COVID-19 were more likely to have cats with detectable viral RNA and SARS-CoV-2 antibodies than households with only one person positive (OR = 4.4; 95% CI = 1.7–11.4) [18]. Access to the outdoors for two hours or more was, on the other hand, associated with a lower risk of infection (OR = 0.17; 95% CI = 0.03–0.96) [19]. These associations suggest that there is a higher risk of infection when there is a higher viral load in cats’ environment. The stronger magnitudes of effect found in other studies could also be due to greater variations in the exposures.

The present study also found that cats with detectable SARS-CoV-2 RNA were more likely to have clinical signs of disease than cats that did not. The clinical signs of disease found in this present study were sneezing, lethargy, vomiting, nasal discharging, coughing and diarrhea, and were similar to previous studies [9, 32]. This finding supports the recommendation for animal health workers taking precautions when in contact with cats showing clinical signs compatible with COVID-19 (respiratory and/or digestive) in a COVID-19 positive household. Similar point estimates of the association between the presence of SARS-CoV-2 antibodies and new clinical signs for dogs (OR = 2.6; 95% CI = 0.8–8.3) and for cats (OR = 2.8; 95% CI = 0.5–15.7) were previously reported [11], though these associations were not statistically significant as the confidence intervals included 1. People with clinical signs associated with COVID-19 disease have also been found to shed the virus for longer periods of time [33–35] and with higher estimated viral loads [36], which could also be the case for cats.

### Limitations and biases

The initial design to recruit households within seven days after the confirmation of a positive human index case was challenging in many ways. The time window for recruiting households and sampling cats was short, resulting in several households that had to be excluded from the study in order to meet the inclusion criteria. This is why this inclusion criteria was relaxed for BC where recruitment was difficult. Also, human respondents in this study were mostly the index case and most of them were suffering from COVID-19 symptoms, therefore affecting their interest in getting involved in the study. While a close partnership between researchers and public health officials allowed the team to sample more cats in Québec, this was more challenging in BC. Recruitment difficulties limited our sample size, which in turn likely affected our capacity to estimate the prevalence of detectable SARS-CoV-2 RNA and seropositivity with precision, and to detect weaker associations with potential risk factors. Established formal and informal relationships have been identified as a key factor for One Health communication channel in a recent study by two of the co-authors [37], and also played a role in our study. Indeed, pre-existing relationships between animal health researchers and public health authorities resulted in smooth and rapid collaboration for the recruitment, which was an important asset and allowed timely sampling of cats after confirmation of the index human case.

Refusal from some households could have also led to selection bias. While no reasons were collected, we hypothesized that households where more humans were sick or had more severe symptoms were less likely to participate. This could have affected both the viral RNA detection and seropositivity prevalence we observed.

Four types of virus variants were during the course of the study [30]. With other SARS-CoV-2 variants, new virus characteristics, such as an improved spillback or spillover capacity, could have led to different results [38]. For example, a previous study demonstrated that cats infected with Omicron shed lower loads of virus and show less clinical signs of disease, compared to cats infected with other variant types [39].

Misclassification biases could also have affected our results. Viral RNA copy number (CT) generally corresponds well to infectious virions, but is distinct from a cell culture-based infectivity assay [40]. RT-qPCR is unlikely to underestimate infectious virions. The ELISA is specific for feline IgG to SARS-Cov-2 spike protein. In acute infection, the immunoglobulins may be dimerized or folded, and may not interact optimally with the recombinant S protein in wells. Therefore, the ELISA may underestimate the amount of IgG. Cross-reactivity is less likely but is not well explored for feline sera. We tried to minimize the impact of transient periods of RNA detection and delayed seropositivity to SARS-CoV-2 antibodies by sampling every cat twice, but this could still have affected our results by underestimating both SARS-CoV-2 RNA detection and seropositivity. The variables obtained via the questionnaires relied on the memory of the human participants and could suffer from recall bias. As owners were not aware of the SARS-CoV-2 status of their cats when the questionnaire was filled, it is unlikely that it would have led to a differential bias in the association found between SARS-CoV-2 RNA detection and clinical signs. No diagnostic test was conducted to confirm the absence of the two most prevalent pathogens involved into upper respiratory tract problems in cats: feline herpesvirus and calicivirus. Therefore, it is possible that those two viruses were involved in the reported clinical signs in those cats. It is also possible that clinical signs could be present in cats infected with SARS-CoV-2 that did not seroconvert, as reported in studies on cats [41, 42] and humans [43, 44].

## Conclusion

This study confirms that SARS-CoV-2 seropositivity of cats in households with a recent human COVID-19 case is frequent, even if the frequency of virus detection on swabs was low. While the number of cats excreting the virus decreased between the first two sampling visits, one cat remained positive at least 32 days after the index case. The results also suggest that recently exposed cats excreting SARS-CoV-2 had a greater prevalence of clinical signs compatible with COVID-19 (respiratory and/or digestive) than cats that did not, which should be taken into consideration if presented in a veterinary facility. These findings contribute to the knowledge that could be used during future outbreaks.

### Electronic supplementary material

Below is the link to the electronic supplementary material.

Supplementary Material 1

Supplementary Material 2

## Data Availability

The data that support the findings of this study are available from the corresponding author upon reasonable request.

## Abbreviations

BC: British Columbia (Canada)
BCCDC: BC Centre for Disease Control
BCI: Bayesian credibility intervals
COVID-19: Coronavirus Disease 2019
DSP: Direction de la santé publique
ELISA: Enzyme-linked immunosorbent assays
HRP: Horseradish peroxidase
INSPQ: Institut national de santé publique du Québec
NOP: Nasal and oropharyngeal
OD: Optical density
PPE: Personal protective equipment
PR: Prevalence ratios
Qc: Québec (Canada)
SARS-CoV-2: Severe acute respiratory syndrome coronavirus 2
STROBE-Vet: Strengthening the Reporting of Observational Studies in Epidemiology – veterinary
