# Supplementary material for: High prevalence of SARS-CoV-2 antibodies and low prevalence of SARS-CoV-2 RNA in cats recently exposed to human cases
Source: BMC Vet Res. 2024 Jul 9;20:304. doi: 10.1186/s12917-024-04150-4 (PMC11232172; doi:10.1186/s12917-024-04150-4)
Supplement: Supplementary file 1 — Supplementary Material 1 [file 12917_2024_4150_MOESM1_ESM.pdf]

Supplementary material: Checklist – S1

The STROBE-Vet statement checklist.

	Item	*STROBE-Vet recommendation*	*Page #*
**Title and Abstract**	1	(a) Indicate that the study was an observational study and, if applicable, use a common study design term	3
		(b) Indicate why the study was conducted, the design, the results, the limitations, and the relevance of the findings	3
**Background / rationale**	2	Explain the scientific background and rationale for the investigation being reported	4
**Objectives**	3	(a) State specific objectives, including any primary or secondary prespecified hypotheses or their absence	4
		(b) Ensure that the level of organization[a] is clear for each objective and hypothesis	4
**Study design**	4	Present key elements of study design early in the paper	5
**Setting**	5	(a) Describe the setting, locations, and relevant dates, including periods of recruitment, exposure, follow-up, and data collection	5-7
		(b) If applicable, include information at each level of organization	5-7
**Participants** [b]	6	(a) Describe the eligibility criteria for the owners/managers and for the animals, at each relevant level of organization	5
		(b) Describe the sources and methods of selection for the owners/managers and for the animals, at each relevant level of organization	5
		(c) Describe the method of follow-up	5
		(d) For matched studies, describe matching criteria and the number of matched individuals per subject (e.g., number of controls per case)	N/A
**Variables**	7	(a) Clearly define all outcomes, exposures, predictors, potential confounders, and effect modifiers. If applicable, give diagnostic criteria	8-9
		(b) Describe the level of organization at which each variable was measured	8-9
		(c) For hypothesis-driven studies, the putative causal-structure among variables should be described (a diagram is strongly encouraged)	

**Data sources / measurement**	8*	(a) For each variable of interest, give sources of data and details of methods of assessment (measurement). If applicable, describe comparability of assessment methods among groups and over time	5-8
		(b) If a questionnaire was used to collect data, describe its development, validation, and administration	7-8
		(c) Describe whether or not individuals involved in data collection were blinded, when applicable	NA
		(d) Describe any efforts to assess the accuracy of the data (including methods used for “data cleaning” in primary research, or methods used for validating secondary data)	
**Bias**	9	Describe any efforts to address potential sources of bias due to confounding, selection, or information bias	
**Study size**	10	(a) Describe how the study size was arrived at for each relevant level of organization	5
		(b) Describe how non-independence of measurements was incorporated into sample-size considerations, if applicable	
		(c) If a formal sample-size calculation was used, describe the parameters, assumptions, and methods that were used, including a justification for the effect size selected	
**Quantitative variables**	11	Explain how quantitative variables were handled in the analyses. If applicable, describe which groupings were chosen, and why	8-9
**Statistical methods**	12	(a) Describe all statistical methods for each objective, at a level of detail sufficient for a knowledgeable reader to replicate the methods. Include a description of the approaches to variable selection, control of confounding, and methods used to control for non-independence of observations	9
		(b) Describe the rationale for examining subgroups and interactions and the methods used	NA
		(c) Explain how missing data were addressed	9
		(d) If applicable, describe the analytical approach to loss to follow-up, matching, complex sampling, and multiplicity of analyses	9
		(e) Describe any methods used to assess the robustness of the analyses (e.g., sensitivity analyses or quantitative bias assessment)	NA
**Participants**	13*	(a) Report the numbers of owners/managers and animals at each stage of study and at each relevant level of organization - e.g., numbers eligible, included in the study, completing follow-up, and analyzed	9-10

		(b) Give reasons for non-participation at each stage and at each relevant level of organization	9-10
		(c) Consider use of a flow diagram and/or a diagram of the organizational structure	
**Descriptive data on exposures and potential confounders**	14*	(a) Give characteristics of study participants (e.g., demographic, clinical, social) and information on exposures and potential confounders by group and level of organization, if applicable	9-10 + Tables 1 and 2
		(b) Indicate number of participants with missing data for each variable of interest and at all relevant levels of organization	Tables 1 and 2
		(c) Summarize follow-up time (e.g., average and total amount), if appropriate to the study design	10
**Outcome data**	15*	(a) Report outcomes as appropriate for the study design and summarize at all relevant levels of organization	10-11
		(b) For proportions and rates, report the numerator and denominator	Tables 1 and 2
		(c) For continuous outcomes, report the number of observations and a measure of variability	10
**Main results**	16	(a) Give unadjusted estimates and, if applicable, adjusted estimates and their precision (e.g., 95% confidence interval). Make clear which confounders and interactions were adjusted. Report all relevant parameters that were part of the model	Tables 1 and 2
		(b) Report category boundaries when continuous variables were categorized	Tables 1 and 2
		(c) If relevant, consider translating estimates of relative risk into absolute risk for a meaningful time period	NA
**Other analyses**	17	Report other analyses done,-such as sensitivity/robustness analysis and analysis of subgroups	NA
**Key results**	18	Summarize key results with reference to study objectives	15
**Strengths and Limitations**	19	Discuss strengths and limitations of the study, taking into account sources of potential bias or imprecision. Discuss both direction and magnitude of any potential bias	13-15
**Interpretation**	20	Give a cautious overall interpretation of results considering objectives, limitations, multiplicity of analyses, results from similar studies, and other relevant evidence	10-12
**Generalizability**	21	Discuss the generalizability (external validity) of the study results	10-12

~~Funding~~ **Transparency**	22	(a) Funding- Give the source of funding and the role of the funders for the present study and, if applicable, for the original study on which the present article is based<br>(b) Conflicts of interest-Describe any conflicts of interest, or lack thereof, for each author<br>(c) Describe the authors' roles- Provision of an authors' declaration of transparency is recommended<br>(d) Ethical approval- Include information on ethical approval for use of animal and human subjects<br>(e) Quality standards-Describe any quality standards used in the conduct of the research	Funding: 17<br><br>Coi: 17<br><br><br>Ethics: 5 and 17

[a] Level of organization recognizes that observational studies in veterinary research often deal with repeated measures (within an animal or herd) or animals that are maintained in groups (such as pens and herds); thus, the observations are not statistically independent. This non-independence has profound implications for the design, analysis, and results of these studies.
[b] The word "participant" is used in the STROBE statement. However, for the veterinary version, it is understood that "participant" should be addressed for both the animal owner/manager and for the animals themselves.
*Give such information separately for cases and controls in case-control studies and, if applicable, for exposed and unexposed groups in cohort and cross-sectional studies.
