# Supplementary material for: High prevalence of SARS-CoV-2 antibodies and low prevalence of SARS-CoV-2 RNA in cats recently exposed to human cases
Source: BMC Vet Res. 2024 Jul 9;20:304. doi: 10.1186/s12917-024-04150-4 (PMC11232172; doi:10.1186/s12917-024-04150-4)
Supplement: Supplementary file 2 — Supplementary Material 2 [file 12917_2024_4150_MOESM2_ESM.pdf]

Supplementary material: Questionnaires – S2

Questionnaires (French version p. 8) developed to collect information on cat- and household-level characteristics and management, and about clinical signs in cats. Questionnaire 1 was used during the first visit within 15 days of the confirmation of the index case, and the Questionnaire 2 was used for subsequent visits.

English version – Questionnaire 1: Transmission dynamics of SARS-CoV-2 in household pets: a one health approach in British Columbia and Quebec

A. Household information

Date interview completed: DD/MM/YYYY Interviewer Name: ___________________

Household ID: _____________________

Who is providing information?
□ Index COVID-19 case □ Other, specify name: ______________ Relationship to index case: ____________________

How many people are currently living in the household? _______

How many pets belong to the household? (include service animals and any animals that primarily live outside if the household members consider them "pets" and interact with them regularly).
□ none □ cats # ____ □ dogs # ____ □ other (list) _____________________________

B. COVID-19 case information

How many people in the household have COVID-19? ______

For the first (index) case in the household: Age: ____ Gender: ☐ Male ☐ Female ☐ Other

Relationship to household pet(s): ______________________

Date of onset of clinical signs: dd/mm/yy ☐ N/A because no clinical signs Date of diagnosis: dd/mm/yy

Clinical signs: ☐ no clinical signs

☐ fever start date: dd/mm/yy end date: dd/mm/yy

☐ cough start date: dd/mm/yy end date: dd/mm/yy

☐ runny nose start date: dd/mm/yy end date: dd/mm/yy

☐ sneezing start date: dd/mm/yy end date: dd/mm/yy

☐ vomiting start date: dd/mm/yy end date: dd/mm/yy

☐ diarrhea start date: dd/mm/yy end date: dd/mm/yy

☐ difficulty breathing or shortness of breath start date: dd/mm/yy end date: dd/mm/yy

☐ loss of smell or taste start date: dd/mm/yy end date: dd/mm/yy

☐ lethargy start date: dd/mm/yy end date: dd/mm/yy

☐ other: describe ____________________ start date: dd/mm/yy end date: dd/mm/yy

Other cases of COVID-19 in the household: ☐ not applicable

Complete this section for each additional human COVID-19 case in the household

COVID-19 case # in house: ____ Age: ____ Gender: ☐ Male ☐ Female ☐ Other

Date of onset of clinical signs: dd/mm/yy ☐ N/A because no clinical signs Date of diagnosis: dd/mm/yy

Clinical signs: ☐ no clinical signs

☐ fever start date: dd/mm/yy end date: dd/mm/yy

☐ cough start date: dd/mm/yy end date: dd/mm/yy

☐ runny nose start date: dd/mm/yy end date: dd/mm/yy

☐ sneezing start date: dd/mm/yy end date: dd/mm/yy

☐ vomiting start date: dd/mm/yy end date: dd/mm/yy

☐ diarrhea start date: dd/mm/yy end date: dd/mm/yy

☐ difficulty breathing or shortness of breath start date: dd/mm/yy end date: dd/mm/yy

☐ loss of smell or taste start date: dd/mm/yy end date: dd/mm/yy

☐ lethargy start date: dd/mm/yy end date: dd/mm/yy

☐ other: describe ____________________ start date: dd/mm/yy end date: dd/mm/yy

C. Household cat information (complete this set of questions for each pet)

1. Cat's name: ______________________________________ Animal ID: _______________________

a. Primary caretaker of [PET NAME]: ________________ ◻ Index COVID-19 case ◻ Other _______________

b. Breed of cat: ______________________________

c. Age of cat (years/months): ____________________________ years or months (circle one)

d. Sex of cat: ☐ Male ☐ Female

.Has [PET NAME] been spayed/neutered: ☐ Yes ☐ No

2. Does [PET NAME] have any current health conditions: ☐ Yes ☐ No

a. If yes, please describe these health conditions or illnesses including when they started:

Condition	Date Started	Medications or supplement for the condition

b. Please describe any other medications or supplements that [PET NAME] takes.

3. On a regular day **on the week before** [index COVID-19 CASE] was diagnosed or first showed clinical signs of COVID-19, how many days a week, how long per day, and what types of interaction/contact did [COVID-19 CASE] usually have with [PET NAME]?

**Types of interaction/contact with pet**	**Number of days per week**	**Average daily duration of interaction/contact (hours or minutes)**
☐ petting		
☐ cuddling (e.g. holding in arms or carrying)		
☐ letting lick face or hands		
☐ cleaning litter box		

☐ brushing or cleaning cat		
☐ cat sleeping on you		
☐ cat rests or sleeps in same bedroom		
☐ cat rests or sleeps in same bed		
☐ feeding by hand (e.g giving treats)		
☐ Other (please describe)		

If there is more than one animal in the house: ☐ no other animals in the household

4. On a regular day **on the week before** [index COVID-19 CASE] was diagnosed or first showed clinical signs of COVID-19, how many days a week, how long per day, and what types of interaction/contact did [PET Name] usually have with other animals?

**Types of interaction/contact between pets**	**Number of days per week**	**Average daily duration of interaction/contact (hours or minutes)**
☐ playing		
☐ resting/sleeping together		
☐ licking/grooming each other		
☐ eating from the same food bowl		

☐ Other (please describe)		

5. On a regular day **<u>in the week after</u>** [COVID-19 CASE] was diagnosed or first showed clinical signs, how many days a week, how long per day and what types of interaction/contact did [COVID-19 CASE] usually have with [PET NAME]?

**Types of interaction/contact with pet**	**Number of days per week**	**Average daily duration of interaction/contact (hours or minutes)**
☐ petting		
☐ cuddling (e.g. holding in arms or carrying)		
☐ letting lick face or hands		
☐ cleaning litter box		
☐ brushing or cleaning cat		
☐ cat sleeping on you		
☐ cat rests or sleeps in same bedroom		
☐ cat rests or sleeps in same bed		
☐ feeding by hand (e.g giving treats)		
☐ Other (please describe)		

6. Was [COVID-19 CASE] wearing any personal protective equipment (e.g. gloves or a cloth face covering)?

☐ Yes ☐ No

a.If yes, please describe: ____________________________

7. **Prior to** [index COVID-19 case] being diagnosed or began showing clinical signs did [PET NAME] reside: ☐ indoors, ☐ outdoors or ☐ both?

a. If both, what percent of time is spent indoors? ____________%

b. If outdoors or both, does [PET NAME] go outside the limit of the household (beyond the yard)? ______________

c. If outdoors, on average how many people does [PET NAME] interact with other than members of the household? _____________

d. Is [PET NAME] allowed anywhere in the house or restricted to certain areas? ________________

e. If restricted, specify where:

8. On a regular day **in the week after** [index COVID-19 case] was diagnosed or began to show clinical signs did [PET NAME] reside: ☐ indoors, ☐ outdoors or ☐ both?

a. If both, what percent of time is spent indoors? ____________%

b. If outdoors or both, does [PET NAME] go outside the limit of the household (beyond the yard)? ______________

c. If outdoors, on average how many people does [PET NAME] interact with other than members of the household? _____________

d. Was [PET NAME] allowed anywhere in the house or restricted to certain areas? ________________

e. If restricted, specify where:

9. On a regular day **in the week after** [index COVID-19 case] was diagnosed or began to show clinical signs, how many days a week, how long per day, and what types of interaction/contact did [PET NAME] usually have with other household pets? ☐ Not applicable, no other household pets

**Types of interaction/contact between pets**	**Number of days per week**	**Average daily duration of interaction/contact (hours or minutes)**
☐ playing together		
☐ resting/sleeping together		
☐ licking/grooming each other		
☐ eating from the same food bowl		
☐ Other (please describe)		

10. Since [COVID-19 case] was diagnosed, has this pet developed any new health condition (mark all that apply and indicate start date and duration of symptoms)?

☐ Coughing Start date: dd/mm/yy End date: dd/mm/yy
☐ Runny nose Start date: dd/mm/yy End date: dd/mm/yy
☐ Sneezing Start date: dd/mm/yy End date: dd/mm/yy
☐ Vomiting Start date: dd/mm/yy End date: dd/mm/yy
☐ Difficulty breathing or shortness of breath Start date: dd/mmm/yy End date: dd/mm/yy
☐ Diarrhea Start date: dd/mm/yy End date: dd/mm/yy
☐ Lethargy Start date: dd/mm/yy End date: dd/mm/yy
☐ Other (please describe): ___________________________

Start date: dd/mm/yy End date: dd/mm/yy

11. Is there any additional information you think we should know about [PET NAME]?

a. If Yes, please describe:

12. Do you consent to the collection of samples from [PET NAME] on 2 occasions, 10 days apart?

You have the right to withdraw consent at any time. The samples that will be collected from [PET NAME] are:

- Blood sample
- Nasal swab
- Oral swab
- Rectal swab

☐ Yes ☐ No

Version française – Questionnaire 1 – Étude COVID chats

Date de l'entrevue:	Nom de l'enquêteur:

Informations sur le foyer

1. Numéro d'identification du foyer:

2. Qui fournit l'information?

□ Cas de COVID-19 confirmé

□ Autre, lien avec le cas confirmé: _____________

3. Combien de personnes vivent dans ce foyer présentement? ______

4. Ce foyer comporte combien d'animaux de compagnie? (incluant les animaux d'assistance et aussi tous les animaux qui vivent principalement à l'extérieur si les membres du foyer les considèrent comme des animaux de compagnie et qu'ils interagissent avec eux régulièrement)

□ aucun

□ chats # ____

□ chiens # ____

□ autre (préciser) ___________________________

Informations sur les personnes infectées par la COVID-19 dans le foyer

5. Combien de personnes dans ce foyer sont positives à la COVID-19? ______

Premier cas confirmé [CAS DE COVID-19 INDEXÉ] ***- <u>Si plus d'un cas confirmé : Compléter à nouveau les questions 7 à 11 pour chacun des cas.</u>***

6. Quelle est l'année de naissance de [CAS DE COVID-19 INDEXÉ]? _________

7. Son genre?

☐ Homme

☐ Femme

☐ Autre

8. La date de son diagnostic (confirmation du test positif)?:

9.1 Êtes-vous vaccinés contre la COVID-19 ? :

 Oui, 1 dose

 Oui, 2 doses

 Non

Précisez le type de vaccin :

10. [CAS DE COVID-19 INDEXÉ] a-t-il eu des symptômes qui peuvent être attribuables à la COVID-19 jusqu'à présent?

 Aucun signe clinique

 Fièvre - Date de début: jj/mmm/aa Date de fin: jj/mmm/aa

 Toux - Date de début: jj/mmm/aa Date de fin: jj/mmm/aa

 Difficultés respiratoires ou essoufflement - Date de début: jj/mmm/aa Date de fin: jj/mmm/aa

 Perte de l'odorat et du goût - Date de début: jj/mmm/aa Date de fin: jj/mmm/aa

 Fatigue intense - Date de début: jj/mmm/aa Date de fin: jj/mmm/aa

 Diarrhée ou vomissements - Date de début: jj/mmm/aa Date de fin: jj/mmm/aa

 Autres (veuillez préciser): _________________ - Date de début: jj/mmm/aa Date de fin: jj/mmm/aa

11. *Le jour de l'échantillonnage* : [CAS DE COVID-19 INDEXÉ] a-t-il des symptômes attribuables à la COVID-19 aujourd'hui?

 Aucun signe clinique

 Fièvre

 Toux

 Difficultés respiratoires ou essoufflement

 Perte de l'odorat et du goût

 Fatigue intense

 Diarrhée ou vomissements

 Autres (veuillez préciser): __________________________

Information sur le chat du foyer ***(compléter ces questions pour chaque chat)***

12. Nu. d'identification de l'animal:

13. Quel est le nom de l'animal?

14. Son âge (ans/mois):

15. Son sexe ?: ☐ Mâle ☐ Femelle

16. Sa race?

17. Est-ce que [NOM DE L'ANIMAL] est stérilisé?: ☐ Oui ☐ Non

18. Est-ce que [NOM DE l'ANIMAL] a un ou des problème(s) de santé connu(s): ☐ Oui ☐ Non

Si oui, veuillez décrire ce(s) problème(s) de santé ou maladie(s), incluant la date de leur début et tout médicament ou supplément actuellement utilisé pour traiter ce problème.

Problème de santé	Date du début	Médicament ou supplément pour traiter ce problème

19. Veuillez mentionner tout autre médicament ou supplément que vous donnez à [NOM DE L'ANIMAL].

20. Au cours des 2 dernières semaines, quel % de son temps [NOM DE L'ANIMAL] a-t-il passé à l'extérieur? ____________%.

Section portant sur les 7 jours <u>précédant</u> le diagnostic positif de [CAS DE COVID-19 INDEXÉ]

21. **<u>Au cours des 7 jours précédant le diagnostic positif</u>** de [CAS DE COVID-19 INDEXÉ], combien y a-t-il eu de jours où il y a eu ce type de contacts avec [NOM DE L'ANIMAL]?

	Nombre de jours parmi les 7 jours
Caresser ou prendre l'animal dans ses bras	
Prendre l'animal dans ses bras	
Laisser l'animal lui lécher le visage ou les mains	
Nettoyer la litière de l'animal	

Brossez ou nettoyez l’animal	
Laissez le chat dormir sur soi	
Laisser le chat passer du temps dans la même pièce	
Nourrir à la main (ex. donner des gâteries)	
Autre contact (précisez; ex. donner de la nourriture de table qu’une personne infectée aurait touché) :	

Si plus d’un animal dans le foyer :

23. **Au cours des 7 jours précédant le diagnostic positif** de [CAS DE COVID-19 INDEXÉ], combien y a-t-il eu de jours où [NOM DE L’ANIMAL] interagissait avec vos autres animaux de compagnie?

	Nombre de jours parmi les 7 jours
Jouer ensemble	
Dormir/se reposer ensemble	
Lavage et léchage mutuel	
Contact avec l’urine ou les fèces des autres animaux	
Partager de la nourriture dans un même bol (en même temps? Oui / Non)	
Autre contact (précisez) :	

25. Est-ce que [CAS DE COVID-19] a changé le type de contact qu’il a avec [NOM DE L’ANIMAL] **suivant son diagnostic positif?**

Si Oui : poursuivre

Si Non : sauter à la question 28

→ 28. Pendant ces interactions, [CAS DE COVID-19 INDEXÉ] portait-il alors un équipement de protection individuelle (ex. gants ou couvre-visage)?

☐ Oui ☐ Non

Si oui, veuillez préciser:

29. Avant ces interactions, [CAS DE COVID-19 INDEXÉ] se lave-t-il toujours les mains?

 Oui Non
Si oui, veuillez préciser:

30. **Depuis la confirmation du diagnostic** de [CAS DE COVID-19 INDEXÉ], est-ce que [NOM DE L'ANIMAL] est libre de circuler à l'extérieur?
 Oui Non
Si oui, veuillez préciser:

31. **Depuis la confirmation du diagnostic** de [CAS DE COVID-19 INDEXÉ], est-ce que [NOM DE L'ANIMAL] est libre de circuler dans la maison ou est-il confiné à certaines parties de la maison?
S'il est confiné, spécifier l'endroit: ________________

32. **Depuis la confirmation du diagnostic** de [CAS DE COVID-19 INDEXÉ], est-ce que [NOM DE L'ANIMAL] a accès à la cuvette des toilette (pour s'abreuver par exemple)?
 Oui Non
Si oui, veuillez préciser:

33. **Depuis la confirmation du diagnostic** de [CAS DE COVID-19 INDEXÉ], est-ce que [NOM DE L'ANIMAL] a développé de nouveaux problèmes de santé (cocher tout ce qui s'applique et noter la date de début, de fin et la durée des signes)?

Toux	Date de début: jj/mmm/aa	Date de fin: jj/mmm/aa
Écoulements nasaux	Date de début: jj/mmm/aa	Date de fin: jj/mmm/aa
Difficultés respiratoires/essoufflement	Date de début: jj/mmm/aa	Date de fin: jj/mmm/aa
Éternuements	Date de début: jj/mmm/aa	Date de fin: jj/mmm/aa
Vomissements	Date de début: jj/mmm/aa	Date de fin: jj/mmm/aa
Diarrhée	Date de début: jj/mmm/aa	Date de fin: jj/mmm/aa
Faiblesse (léthargie)	Date de début: jj/mmm/aa	Date de fin: jj/mmm/aa
Autre (veuillez décrire): _____________	Date de début: jj/mmm/aa	Date de fin: jj/mmm/aa

34. Y a-t-il autre chose que nous devrions savoir au sujet de [NOM DE L'ANIMAL]? (Est-ce que l'animal a été en contact avec des gens de l'extérieur, des gens vulnérables, fait de la zoothérapie normalement, autres?)

Questionnaire 2 – Étude COVID chats

Date de l'entrevue:	Nom de l'enquêteur:

Informations sur le foyer

1. Numéro d'identification du foyer:

2. Qui fournit l'information?

□ Cas de COVID-19 confirmé

□ Autre, lien avec le cas confirmé: _____________

3. **<u>Depuis notre première visite, y</u>** a-t-il eu des changements dans le nombre de personnes qui vivent dans ce foyer présentement? __ _____

4. **<u>Depuis notre première visite</u>** , y a-t-il eu des changements dans le nombre d'animaux de compagnie qui vivent dans ce foyer? (incluant les animaux d'assistance et aussi tous les animaux qui vivent principalement à l'extérieur si les membres du foyer les considèrent comme des animaux domestiques et qu'ils interagissent avec eux régulièrement)

□ aucun

□ chats # ____

□ chiens # ____

□ autre (préciser) ___________________________

Informations sur les personnes infectées par la COVID-19 dans le foyer

5. **<u>Depuis notre première visite</u>**, y a-t-il des personnes additionnelles dans ce foyer qui ont reçu un diagnostic de COVID-19? _____

Si non, compléter seulement pour le premier cas confirmé (cas index) et sauter les questions 7 à 11)

6. **<u>Depuis notre première visite</u>** [CAS DE COVID-19 INDEXÉ] a-t-il eu des symptômes qui peuvent être attribuable à la COVID-19 à la COVID-19?

☐ Aucun signe clinique

☐ Fièvre - Date de début: jj/mmm/aa Date de fin: jj/mmm/aa

☐ Toux - Date de début: jj/mmm/aa Date de fin: jj/mmm/aa

☐ Difficultés respiratoires ou essoufflement - Date de début: jj/mmm/aa Date de fin: jj/mmm/aa

☐ Perte de l'odorat et du goût - Date de début: jj/mmm/aa Date de fin: jj/mmm/aa :

☐ Fatigue intense - Date de début: jj/mmm/aa Date de fin: jj/mmm/aa

☐ Diarrhée ou vomissements - Date de début: jj/mmm/aa Date de fin: jj/mmm/aa

☐ Autres (veuillez préciser): ___________ - Date de début: jj/mmm/aa Date de fin: jj/mmm/aa

7. *Le jour de l'échantillonnage* : [CAS DE COVID-19 INDEXÉ] a-t-il des symptômes attribuables à la COVID-19 aujourd'hui?

☐ Aucun signe clinique

☐ Toux

☐ Écoulements nasaux

☐ Éternuements

☐ Difficultés respiratoires ou essoufflement

☐ Diarrhée

☐ Vomissements

☐ Léthargie

☐ Autres (veuillez préciser): ___________________________

Information sur le chat du foyer *(compléter ces questions pour chaque chat)*

8. **<u>Depuis notre dernière visite</u>**, est-ce que les personnes infectées par la COVID-19 ont changé la fréquence et le type de contact avec [NOM DE L'ANIMAL]?

☐ Oui

☐ Non

→ 9. **<u>Depuis notre visite, pendant ces interactions</u>**, [CAS DE COVID-19] portait-il un équipement de protection individuelle (ex. gants ou couvre-visage)?

☐ Oui ☐ Non

Si oui, veuillez préciser:

10. **Depuis notre dernière visite, avant ces interactions,** [CAS DE COVID-19 INDEXÉ] se lave-t-il toujours les mains?

 Oui Non

Si oui, veuillez préciser:

11. **Depuis notre dernière visite**, est-ce que [NOM DE L'ANIMAL] est libre de circuler à l'extérieur?

 Oui Non

Si oui, veuillez préciser:

12. **Depuis notre dernière visite**, est-ce que [NOM DE L'ANIMAL] est libre de circuler dans la maison ou est-il confiné à certaines parties de la maison? ____________________S'il est confiné, spécifier l'endroit: ______________

13. **Depuis notre dernière visite**, est-ce que [NOM DE L'ANIMAL] a accès à la cuvette des toilette (pour s'abreuver par exemple)?

 Oui Non

Si oui, veuillez préciser:

Si plus d'un animal dans le foyer :

14. **Depuis notre dernière visite**, combien y a-t-il eu de jours où [NOM DE L'ANIMAL] interagissait avec les autres animaux domestiques de la maison pour (…)?

	Nombre de jours parmi les 7 jours
Jouer ensemble	
Dormir/se reposer ensemble	
Lavage et léchage mutuel	
Contact avec l'urine ou les fèces des autres animaux	

Partager de la nourriture dans un même bol (en même temps? Oui / Non)	
Autre contact (précisez) :	

→ 15. **<u>Depuis notre dernière visite</u>**, est-ce que [NOM DE L'ANIMAL] a développé de nouveaux problèmes de santé (cocher tout ce qui s'applique et noter la date de début, de fin et la durée des signes)?

☐ Toux Date de début: jj/mmm/aa Date de fin: jj/mmm/aa
☐ Écoulements nasaux Date de début: jj/mmm/aa Date de fin: jj/mmm/aa
☐ Difficultés respiratoires/essoufflement Date de début: jj/mmm/aa Date de fin: jj/mmm/aa
☐ Éternuements Date de début: jj/mmm/aa Date de fin: jj/mmm/aa
☐ Vomissements Date de début: jj/mmm/aa Date de fin: jj/mmm/aa
☐ Diarrhée Date de début: jj/mmm/aa Date de fin: jj/mmm/aa
☐ Faiblesse (léthargie) Date de début: jj/mmm/aa Date de fin: jj/mmm/aa
☐ Autre (veuillez décrire): _____________ Date de début: jj/mmm/aa Date de fin: jj/mmm/aa

16. Y a-t-il autre chose que nous devrions savoir ou qui ont changé depuis notre dernière visite au sujet de [NOM DE L'ANIMAL]?
